# THE EXPLORATION OF CORRELATES OF LONELINESS AMONG COMMUNITY-DWELLING OLDER PEOPLE’S TECHNOLOGY LITERACY

**DOI:** 10.1093/geroni/igac059.2198

**Published:** 2022-12-20

**Authors:** Suk-Young Kang, Jeungkun Kim, Antonina Poplawski

**Affiliations:** Binghamton University, State University of New York, Binghamton, New York, United States; Kangnam University, Youngin, Kyonggi-do, Republic of Korea; Binghamton University, Binghamton, New York, United States

## Abstract

Social isolation is a major public health risk among older adults, even considered to be as harmful as smoking. The aim of this study was to explore the benefits of digital literacy and the correlates related to levels of loneliness on older people.Mail-survey data were collected from community-dwelling adults over the age of 65. There were 192 total participants, with information from 180 people being included in the analysis.The six-item De Jong Loneliness Scale was used to measure levels of loneliness. Among the 180 participants, the mean was 2.02 (SD = 1.68).Regression analysis revealed that the equation explained 48.2% of variance (adjusted R-squared = .446) in levels of loneliness (F (11,162) = 13.682, p <.000). Variance inflation factor (VIF) values were smaller than 10, indicating that multicollinearity among the correlates was not an issue. Correlates of levels of loneliness included aging anxiety, depression, social network, and self-evaluated technology literacy with computer software programs compared to peers. Results confirmed the correlates related to loneliness among older people identified in previous studies. This shows quality of life related to mental health may be improved by a positive attitude towards technology use.This study further found that digital literacy affects loneliness when controlling for income, depression, level of network, and physical health.Participants who could increase their digital literacy are interested in more opportunities on how to use new technology. Policy changes that allow for lifelong learning options could support this, while also decreasing levels of social isolation and loneliness.

